# Supplementary material for: Neurons define non-myelinated axon segments by the regulation of galectin-4-containing axon membrane domains
Source: Sci Rep. 2017 Sep 25;7:12246. doi: 10.1038/s41598-017-12295-6 (PMC5612983; doi:10.1038/s41598-017-12295-6)
Supplement: Supplementary file 1 — Supplementary information [file 41598_2017_12295_MOESM1_ESM.pdf]

Supplementary material for:

Neurons define non-myelinated axon segments by the regulation of galectin-4-containing axon membrane domains

Natalia Díez-Revuelta[1] Alonso M. Higuero[#], Silvia Velasco[1], María Peñas-de-la-Iglesia[1], Hans-Joachim Gabius[2], José Abad-Rodríguez[1]

[1]*Membrane Biology and Axonal Repair Laboratory. Hospital Nacional de Parapléjicos (SESCAM), Finca La Peraleda s/n, E-45071 Toledo, Spain.*

[2]*Institut für Physiologische Chemie, Tierärztliche Fakultät, Ludwig-Maximilians-Universität, Veterinärstr. 13, D-80539 München, Germany.*

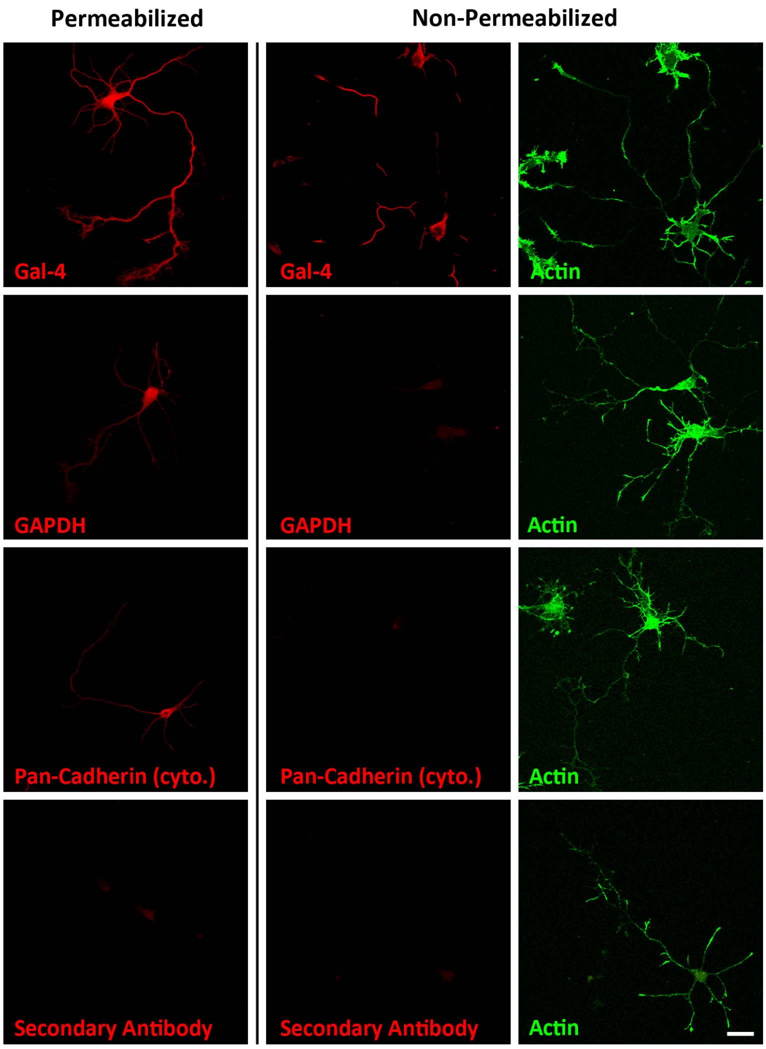

Supplementary Figure S1. Intracellular antigens are not labelled by their specific antibodies in non-permeabilized conditions.

(**A**) Hippocampal neurons (72 hiv) were fixed and permeabilized with 0.1% Triton X-100 in PBS during 5 minutes prior to blocking and immunolabelling (Permeabilized). Simultaneously, hippocampal neurons (72 hiv) were also fixed, blocked and immunolabelled but the permeabilization step was omitted (Non-Permeabilized). Phalloidin-FITC (green, third column) was used to stain actin and visualize the cells under non-permeabilized conditions. Intracellular Gal-4 (red, top row) is detected throughout the whole cell under permeabilized conditions. However, cell surface bound Gal-4 is detected only in discrete segments along the plasma membrane when cells are not permeabilized. Under

the same conditions, GAPDH (red, second row) is detected with a specific antibody (Chemicon MAB374, clone 6C5) throughout the whole neuron, but mainly in the cell body, when cells were permeabilized. In contrast, under non-permeabilized conditions, GAPDH is not detected. Moreover, the cytoplasmic tails of transmembrane cadherins (red, third row) are also not labelled when cells are not permeabilized. This same antibody (Sigma C1821, clone CH-19) detects neuronal cadherins when cells are permeabilized. Finally, fluorophore-conjugated secondary antibodies do not label neither permeabilized nor non-permeabilized cells in the absence of a primary antibody (Secondary Antibody, red, bottom row). Microscope settings were identical during image acquisition of permeabilized vs non-permeabilized cells. (Scale bar 25 μm).

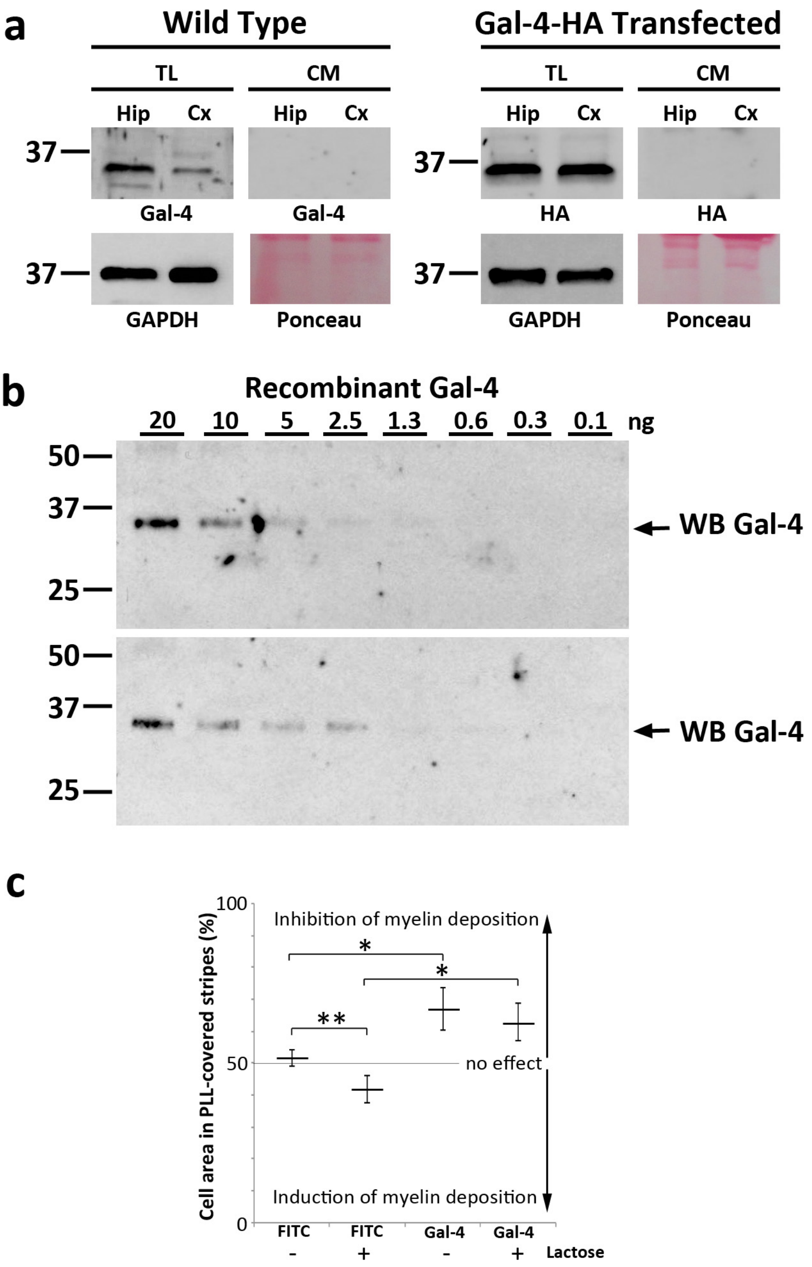

Supplementary Figure S2. Gal-4 expression levels in hippocampal and cortical cell lysates and conditioned media. Effect of lactose on Gal-4-impaired myelin deposition.

(a) Wild type and Gal-4-HA-transfected hippocampal (Hip) and cortical (Cx) neurons were cultured for 7 div. Conditioned media (CM) were collected and cells were lysed (TL) to determine the expression levels of endogenous galectin 4 (Gal-4) and exogenously expressed HA-tagged Gal-4 (HA). Gal-4 and Gal-4-HA are readily detected in total cell lysates of both hippocampal and cortical neurons. However, we were not able to detect neither endogenous nor

exogenously expressed Gal-4 in the conditioned media. A GAPDH immunoblot is shown as a loading control for the total cell lysates, while a Ponceau S staining of the transferred nitrocellulose membrane is shown for the conditioned media. (b) Decreasing amounts of human recombinant Gal-4 were loaded onto polyacrylamide gels (in duplicate) to determine the sensitivity of Gal-4 detection by Western Blot analysis with our Gal-4 antibody. After SDS-PAGE electrophoresis, transfer and immunoblotting, we were able to establish our detection limit in approximately 1 ng of protein. In consequence, in our cell culture conditions (4 ml of CM), Gal-4 secreted to medium would have to be over a concentration of 6.7 picomolar to be detected. (c) OLGs were allowed differentiate for 7-9 div on coverslips with parallel stripes covered with FITC, FICT (1 hour, RT), FITC and recombinant Gal-4, or FITC and recombinant Gal-4 incubated with 25 mM lactose in the same conditions. FITC (brighter stripes) was used together with galectins to evidence galectin-covered stripes. Cells were immunolabeled for MBP to visualize myelin. Quantitative analysis of the repulsive effect exerted by Gal-4 on mature OLGs is shown as the percentage of MBP-positive area on galectin-free stripes (covered with PLL alone). 50% indicates no preference of myelin deposition (no effect), while over and below 50% indicates inhibition and induction of myelin deposition, respectively (arrows in graph). The presence of lactose tends to partially revert Gal-4 inhibition of myelin deposition, although the differences are not statistically significant. It is important to note that in these conditions lactose produce a slight but significant stimulation of myelin deposition when compared to FITC alone control. Values are means +/- s.e.m. of three experiments (n=20) (*p<0.001, **p<0.05; two-tailed, student's *t*-test).

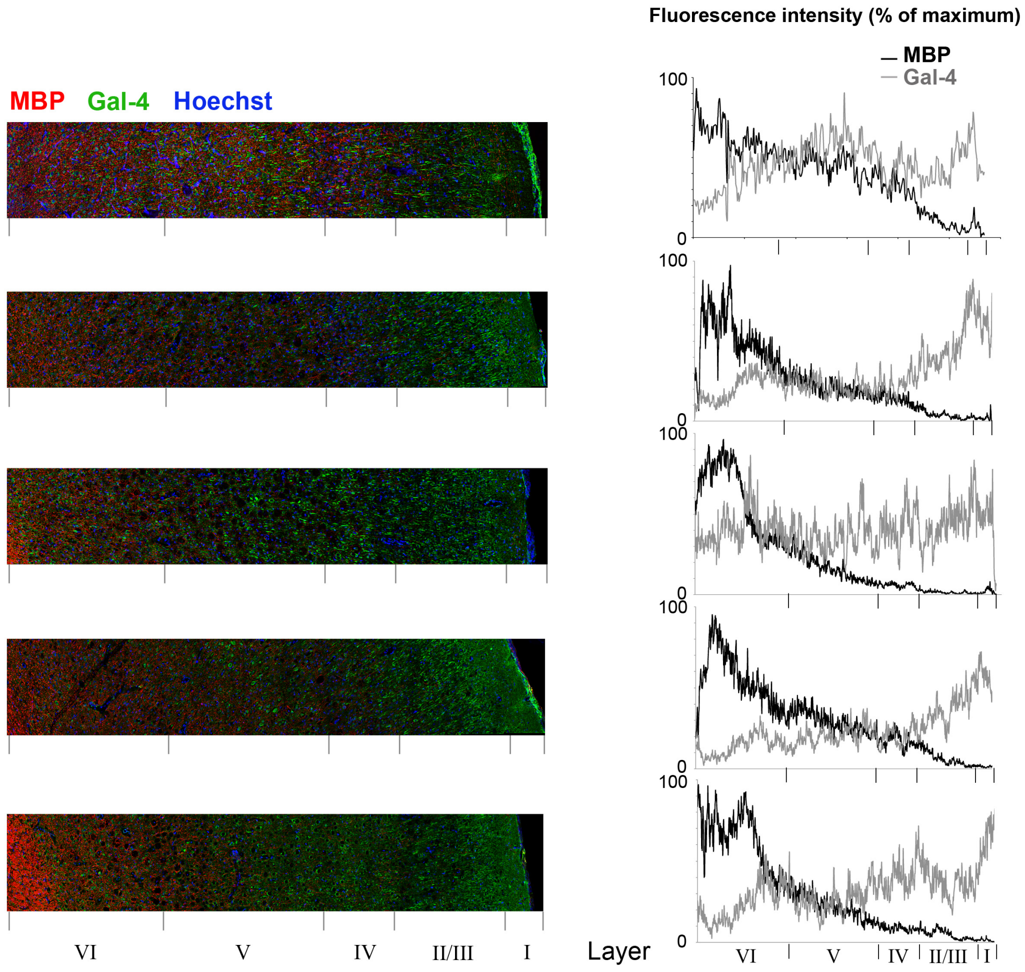

Supplementary Figure S3. Gal-4 distribution along the brain cortex inversely correlates with myelin.

MBP (red) and Gal-4 (green) fluorescence intensities measured in P30 rat brain cortices (n=5). Values are normalized as percentage of the maximal intensity obtained for each channel. In graphs on the right, profiles are plotted from inner to outer brain cortex layers (layer VI to layer I; MBP black line, Gal-4 gray line).

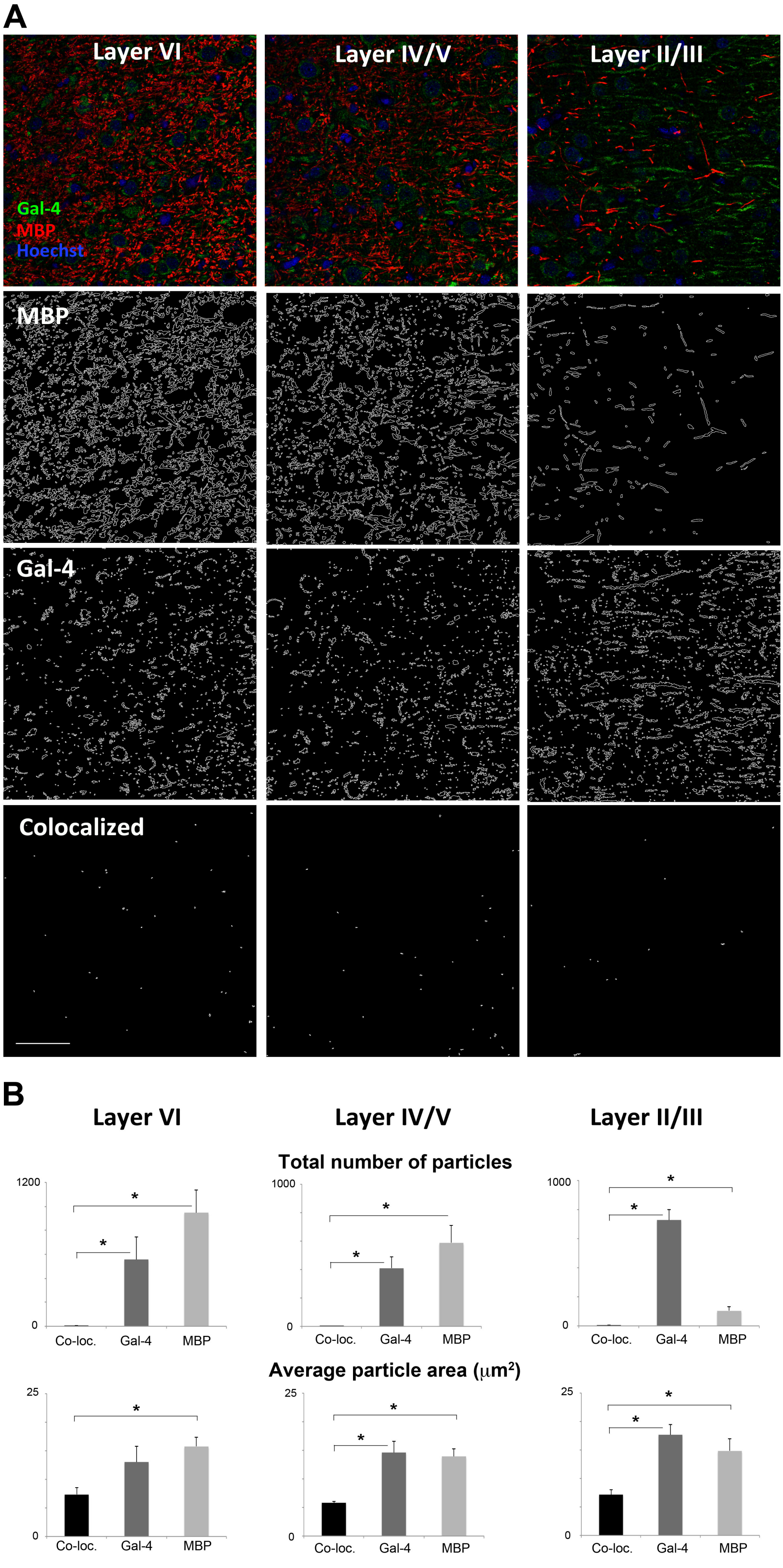
A
Layer VI
Layer IV/V
Layer II/III
Gal-4
MBP
Hoechst
MBP
Gal-4
Colocalized
B
Layer VI
Layer IV/V
Layer II/III
Total number of particles
1200
0
1000
0
1000
0
Co-loc.
Gal-4
MBP
Average particle area (μm2)
25
0
Co-loc.
Gal-4
MBP

Supplementary Figure S4. Co-localization analysis between Gal-4 and MBP in rat brain cortex.

(A) Single confocal planes from selected regions within the somatosensory cortex (cortical layers VI, IV/V and II/III; upper panels left to right) of a representative P30 rat brain (5 brains were analysed). The sections were immunolabeled for MBP (red) and Gal-4 (green), while nuclei were stained with Hoechst (blue). MBP and Gal-4 positive particle maps (middle panels) were analyzed to determine their degree of co-localization (See methods section for details). Co-localizing particle map is shown in the lower panels (Colocalized). (B) Quantification of average number and area of co-localizing particles (black bars in the bar graphs) indicate that co-localization level is low, and that the average size of co-localizing particles is significantly smaller compared to Gal-4 (gray bars) or MBP (pale gray bars) particles. These results corroborate a similar analysis showed in Figure 5E, performed on the maximum projections (combination of all confocal planes) of the same tissue samples. Values are means + s.e.m.; n=5; *p < 0,001, two-tailed Student's t-test.

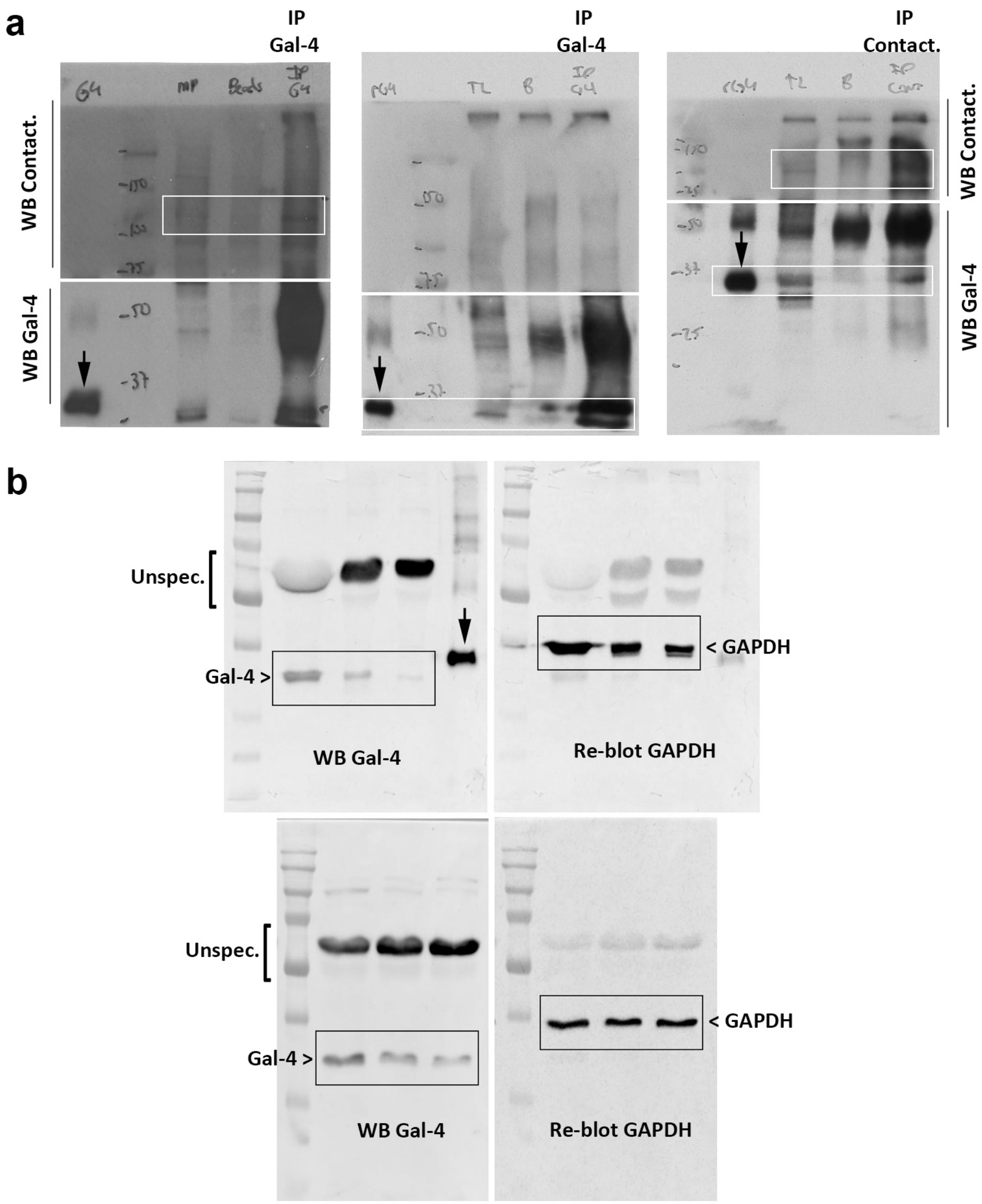

Supplementary Figure S5. Source full-length Western blots used in cropped panel in figures 3 and 4.

(a) PC12 cell cultures were reversibly crosslinked, lysed, and total extracts (TL) were used to immunoprecipitate Gal-4 or contactin. The immunoprecipitated complexes were separated by SDS-PAGE and detected by WB. The nitrocellulose filters were cut

below the 75-kDa molecular weight marker and the upper parts were blotted to visualize contactin, while the lower parts were blotted to visualize Gal-4. Recombinant Gal-4 (arrows), and incubations with protein-A or –G Sepharose beads in the absence of antibodies (Beads) were used as controls. These are crude images from film exposure. To improve visibility of the cropped parts (white boxes) shown in figure 3, contrast and brightness optimizations were applied (no filters or other manipulations were carried out).

(b) WB for total Gal-4 expression in extracts of hippocampal (upper blots) and cortical (lower blots) neurons at 3, 7 and 14 days in culture (from left to right in delineated boxes). Recombinant human Gal-4 (arrows) was used as reference. After blotting for Gal-4, filters were re-blotted for GADPH as loading control. These are the crude images used to perform densitometric analyses. Slight contrast and brightness optimizations were applied to improve visibility of the cropped parts (boxes) shown in figure 4 (no filters or other manipulations were carried out).

Supplementary Video. 3D-reconstruction of in vitro myelinated axon segment flanked by G4Ds.

Confocal Z-planes obtained from selected fields of cortical neuron/oligodendrocyte co-cultures (see Methods for details) were transformed using the 3D-viewer plugin (surface option) of Fiji software (NIH) in order to simulate opaque surfaces of myelin (green channel) and Gal-4 (red channel). Resulting images of 3D-reconstructions were then rotated with respect to a central, vertical axis, using the rotate option of the same plugin, and mounted to generate videos of the red channel alone, and of the mixed channels in parallel.

In this video, the view from above the culture surface (indicated in the video as "Top") shows a myelin sheet (green) covering a long axon tract. Importantly, the view from bellow the culture surface (indicated in the video as "Bottom"), shows that from that tract, the only portion of axon devoid of Gal-4 (red) is completely wrapped by myelin, thus covered from the top and from the bottom. In contrast, axon segments maintaining Gal-4 expression are not myelinated, even if the myelin runs over them.
